# Cardiac magnetic resonance defines mechanisms of sex-based differences in outcomes following cardiac resynchronization therapy

**DOI:** 10.3389/fcvm.2022.1007806

**Published:** 2022-09-15

**Authors:** Derek J. Bivona, Srikar Tallavajhala, Mohamad Abdi, Pim J. A. Oomen, Xu Gao, Rohit Malhotra, Andrew Darby, Oliver J. Monfredi, J. Michael Mangrum, Pamela Mason, Sula Mazimba, Michael Salerno, Christopher M. Kramer, Frederick H. Epstein, Jeffrey W. Holmes, Kenneth C. Bilchick

**Affiliations:** ^1^Department of Medicine, University of Virginia Health System, Charlottesville, VA, United States; ^2^Department of Biomedical Engineering, University of Virginia, Charlottesville, VA, United States; ^3^Department of Biomedical Engineering, University of California, Irvine, Irvine, CA, United States; ^4^Department of Medicine, Northwestern University, Chicago, IL, United States; ^5^Department of Medicine and Radiology, Stanford University, Palo Alto, CA, United States; ^6^Department of Radiology and Medical Imaging, University of Virginia Health System, Charlottesville, VA, United States; ^7^Department of Medicine, Surgery, and Biomedical Engineering, University of Alabama at Birmingham, Birmingham, AL, United States

**Keywords:** sex differences, magnetic resonance imaging, heart failure, cardiac resynchronization therapy, **implantable cardioverter** defibrillator

## Abstract

**Background:**

Mechanisms of sex-based differences in outcomes following cardiac resynchronization therapy (CRT) are poorly understood.

**Objective:**

To use cardiac magnetic resonance (CMR) to define mechanisms of sex-based differences in outcomes after CRT and describe distinct CMR-based phenotypes of CRT candidates based on sex and non-ischemic/ischemic cardiomyopathy type.

**Materials and methods:**

In a prospective study, sex-based differences in three short-term CRT response measures [fractional change in left ventricular end-systolic volume index 6 months after CRT (LVESVI-FC), B-type natriuretic peptide (BNP) 6 months after CRT, change in peak VO_2_ 6 months after CRT], and long-term survival were evaluated with respect to 39 baseline parameters from CMR, exercise testing, laboratory testing, electrocardiograms, comorbid conditions, and other sources. CMR was also used to quantify the degree of left-ventricular mechanical dyssynchrony by deriving the circumferential uniformity ratio estimate (CURE-SVD) parameter from displacement encoding with stimulated echoes (DENSE) strain imaging. Statistical methods included multivariable linear regression with evaluation of interaction effects associated with sex and cardiomyopathy type (ischemic and non-ischemic cardiomyopathy) and survival analysis.

**Results:**

Among 200 patients, the 54 female patients (27%) pre-CRT had a smaller CMR-based LVEDVI (*p* = 0.04), more mechanical dyssynchrony based on the validated CMR CURE-SVD parameter (*p* = 0.04), a lower frequency of both late gadolinium enhancement (LGE) and ischemic cardiomyopathy (*p* < 0.0001), a greater RVEF (*p* = 0.02), and a greater frequency of LBBB (*p* = 0.01). After categorization of patients into four groups based on cardiomyopathy type (ischemic/non-ischemic cardiomyopathy) and sex, female patients with non-ischemic cardiomyopathy had the lowest CURE-SVD (*p* = 0.003), the lowest pre-CRT BNP levels (*p* = 0.01), the lowest post-CRT BNP levels (*p* = 0.05), and the most favorable LVESVI-FC (*p* = 0.001). Overall, female patients had better 3-year survival before adjustment for cardiomyopathy type (*p* = 0.007, HR = 0.45) and after adjustment for cardiomyopathy type (*p* = 0.009, HR = 0.67).

**Conclusion:**

CMR identifies distinct phenotypes of female CRT patients with non-ischemic and ischemic cardiomyopathy relative to male patients stratified by cardiomyopathy type. The more favorable short-term response and long-term survival outcomes in female heart failure patients with CRT were associated with lower indexed CMR-based LV volumes, decreased presence of scar associated with prior myocardial infarction and ICM, and greater CMR-based dyssynchrony with the CURE-SVD.

## Introduction

Cardiac resynchronization therapy (CRT), a pacing therapy used to treat chronic systolic heart failure (HF) and wide QRS complexes (1, 2), has been shown to improve left ventricular function, New York Heart Association (NYHA) functional class, HF hospitalization rates, and overall survival (3–8); however, non-response to CRT is a significant challenge, as 30–50% of patients do not meet standard response criteria for this therapy (9). Prior studies have noted differences in cardiac structural characteristics of male and female heart failure patients undergoing CRT such as left ventricular (LV) size (10–14) with demonstration, for example, of better clinical outcomes in the MIRACLE (Multicenter InSync Randomized Clinical Evaluation) study (15) and a sub-study of the MADIT-CRT (Multicenter Automatic Defibrillator Implantation Trial with Cardiac Resynchronization Therapy) (16). An important limitation of prior work in this area is that the studies have been based largely on echocardiography. In this regard, analyses based on well-curated datasets with cardiac magnetic resonance (CMR) data, response findings based on exercise peak VO_2_ data (17, 18) and laboratory data such as B-type natriuretic peptide (BNP) (19), and long-term clinical outcomes are needed to provide a better understanding of the mechanisms associated with heart failure response in men and women.

In the present study, we hypothesized that scar presence associated with prior myocardial infarction, LV/RV size and function by CMR cine imaging, and the validated CURE-SVD CMR strain parameter (20) calculated using cine displacement encoding with stimulated echoes (DENSE) would be among the key explanatory parameters from CMR to provide an understanding of sex-based differences in CRT response. Furthermore, we hypothesized that cardiomyopathy etiology (non-ischemic versus ischemic) would also be important in defining mechanistic differences in female and male patients not only on the basis of the prevalence of each etiology in men and women, but also in relation to differences in each cardiomyopathy type in women versus men. In this sense, we also aimed to define CMR phenotypes among CRT candidates for female non-ischemic cardiomyopathy, male non-ischemic cardiomyopathy, female ischemic cardiomyopathy, and male ischemic cardiomyopathy. These hypotheses and aims were addressed using a single-center dataset with all the features described above.

## Materials and methods

### Study design, cohort selection, and informed consent

**FIGURE 1:**
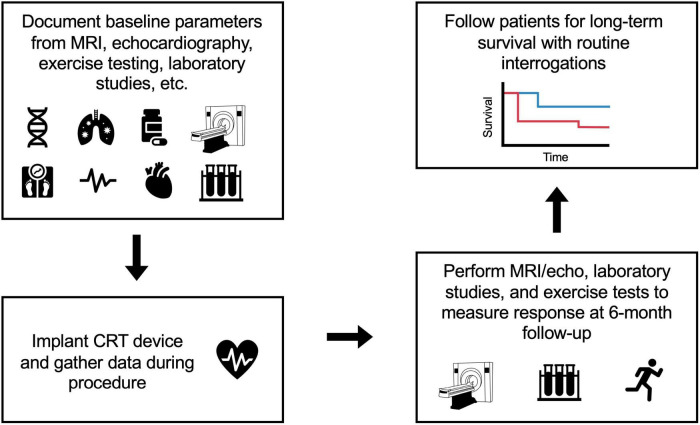
Flow diagram of the study design. Patients were enrolled over approximately a 10-year period from 2011 to 2021. Before CRT device implantation, the baseline parameters of the patients were recorded based on findings from MRI, echocardiography, electrocardiograms, exercise testing, and laboratory studies. Additional parameters (such as QLV which indicates late activation of LV pacing cite) were gathered during the CRT procedure. Six months after CRT, patients received follow-up MRI/echocardiograms, laboratory studies, and exercise testing to calculate response measures. Finally, patients were followed for long-term survival with routine interrogations.

This research was approved by the Institutional Review Board for Human Subjects Research at the University of Virginia (UVA) and conducted over a 10-year period from 2011 to 2021 during which all patients provided informed consent. Inclusion criteria were LVEF 35% or less, New York Heart Association (NYHA) functional class II-IV, QRS > 120 ms, and a class I or class II indication for CRT based on AHA/ACC/HRS guidelines. Additionally, all patients received CRT defibrillators except for one who received a CRT pacemaker. The flow diagram that represents the design of the observational study is shown in Figure 1.

### Baseline patient characteristics

Before CRT implantation at the UVA Health System, patients completed intake forms to document their demographic characteristics, comorbid conditions, and medications; these data were confirmed by cross-checking electronic medical records. Baseline characteristics included age, sex, race, and comorbid conditions (in addition to heart failure). These comorbid conditions included hypertension, atrial fibrillation, chronic kidney disease, diabetes mellitus, prior coronary artery bypass grafting surgery, and ischemic cardiomyopathy (ICM). In this study, ICM was defined as cardiomyopathy associated with prior myocardial infarction and significant contribution of ischemic heart disease to LV dysfunction. Prior infarction was also assessed with late gadolinium enhancement (LGE) on CMR. In the majority of cases, LGE was in an ischemic distribution. Prescribed medications at the time of CRT implantation, including beta-blockers, ACE inhibitor/angiotensin receptor blockers, loop diuretic usage and dosage, digoxin, and statins, were also extracted from the electronic health record. Patients received laboratory studies (including BNP, creatinine, sodium, and hemoglobin), blood pressure assessments, and exercise testing before the CRT procedure. Electrocardiographic data such as QRS duration and bundle branch block morphology were documented from 12-lead ECGs prior to CRT.

### Features recorded at baseline and 6 months after cardiac resynchronization therapy

Cardiopulmonary exercise testing was performed at baseline and again 6 months after CRT for patients able to exercise. The peak VO_2_, VE/VCO_2_ slope, and respiratory exchange ratio were recorded. Echocardiography with standard 2D echocardiographic images were obtained for all patients at baseline and 6 months after CRT, and volumetric measurements indexed for body surface area were calculated using standard methodology (21). CMR examinations were performed for all patients before CRT and for 38% of patients 6 months after CRT. The CMR protocol included steady-state free precession cine imaging, cine DENSE, and LGE. Circumferential strain from cine DENSE was calculated semiautomatically to determine CURE-SVD (range, 0–1, 1 = greatest synchrony). In patients with CMR performed 6 months after CRT, CMR cine imaging was used to calculate the change in LV function, while echocardiographic measurement before and after CRT were used for this purpose in other patients.

### Post-cardiac resynchronization therapy response measures

As CRT response can be assessed in several ways, three different measures of CRT response were recorded at 6 months based on LV function, the neurohormonal axis, and exercise capacity, respectively. With respect to LV function, the fractional change in the LVESVI (LVESVI-FC) was defined as the (post-CRT LVESVI – baseline LVESVI)/baseline LVESVI, such that a more negative number reflected smaller (more favorable) post-CRT LV volumes. Pre-CRT and post-CRT MRIs were used to determine the LVESVI-FC in the 38% of patients who received post-CRT MRIs, while pre-CRT and post-CRT echocardiograms were used to determine the LVESVI-FC in the remaining 62% of patients.

With respect to the neurohormonal axis, the BNP post-CRT was log-transformed and used instead of the absolute BNP levels because it was considered a more meaningful parameter, as BNP values can range from less than 100 to over 5,000 pg/mL. The post-CRT BNP was used rather than a ratio measure based on prior analyses demonstrating the importance of the post-CRT value of the BNP relative to any ratio measure. With respect to exercise capacity, the change in peak oxygen output (Δ peak VO_2_ = VO_2_ post-CRT – VO_2_ pre-CRT) was calculated. These response measures were calculated and reviewed by the data analysts without knowledge of patient outcomes.

### Statistical analysis

#### Missing data

Only 2% of the imaging-based parameters (CURE-SVD and ventricular volumes) gathered before the CRT procedure were missing and were imputed using their respective median values. The change in peak VO_2_ was missing in 20% of patients since some patients had difficulty exercising both before and after CRT. The expectation maximation (EM) algorithm for matrix completion was used to impute these missing measures (22). The Supplementary material describes this imputation technique in more detail.

#### Statistical tests and linear regression with interaction term

With the complete data set, the cohort was stratified into males and females, and statistical tests were performed to identify the sex-based differences in clinical parameters and CMR findings in patients undergoing CRT. Chi-square tests were used to compare discrete variables between the two groups, while *t*-tests were used for comparisons of continuous variables. To assess the effect of sex, cardiomyopathy type, and their interaction on CRT response measures and survival, multivariable linear regression models were constructed with an interaction term. Linear regression models for CURE-SVD along with the three response measures were implemented using the *statsmodels* package in Python.

#### Survival analysis and exploring mechanisms of response

Kaplan–Meier analysis was used to construct survival curves of three stratifications of the data: males vs. females, males with ICM vs. females with ICM, and males with NICM vs. females with NICM. Log-rank tests were used to determine the *p*-values for the differences in survival among the groups, and Cox proportional hazards regression was used to calculate hazard ratios (HRs). The Kaplan–Meier analyses, log-rank tests, and Cox regression were performed using the *lifelines* package in Python. Finally, the data was split into four groups (males with ICM, females with ICM, males with NICM, and females with NICM), and intergroup differences among CURE-SVD, LVEDVI, RVEF, log-transformed pre-CRT BNP, LVESVI-FC, and log-transformed post-CRT BNP were calculated using ANOVA. Tukey tests were run to compare group means following a significant ANOVA.

## Results

### Baseline characteristics of entire cohort

Baseline characteristics for the 200 patients (age 66.1 ± 11.4 years; 27.0% female) are shown in Table 1. The median change in the LVESVI-FC following CRT was –0.18 (interquartile range –0.33 to –0.01). In terms of response, 56.0% of patients had 15% or greater reduction in the LVESVI post-CRT (LVESVI-FC ≤ -0.15). In the entire cohort, the median log-transformed post-CRT BNP level was 2.25 (IQR 1.77–2.77), and the median change in the peak VO_2_ was 0.0 mL/kg/min (IQR –1.0–1.2). During a median follow-up of 3 years, 28 (14.0%) patients died.

**TABLE 1:** Baseline characteristics and CRT response measures of patient cohort and male vs. female.

	Cohort (*N* = 200)	Male (*N* = 146)	Female (*N* = 54)	*P*-value
**Demographics**				
Age, years	67.4 (58.0–74.0)	68.0 (59.9–75.0)	65.9 (56.0–72.0)	0.2
BMI	28.9 (25.4–33.7)	29.3 (25.8–32.9)	28.0 (23.3–36.4)	0.6
Weight (kg)	89.4 (75.1–103.0)	91.6 (80.7–105.2)	75.0 (58.4–95.8)	< 0.0001
Female	54 (27.0)			
**NYHA heart failure class**				0.5
II	73 (36.5)	56 (38.4)	17 (31.5)	
III	126 (63.0)	89 (61.0)	37 (68.5)	
IV	1 (0.50)	1 (0.6)	0 (0)	
Race				0.02
Black	27 (13.5)	14 (9.6)	13 (24.1)	
White/other	173 (86.5)	132 (90.4)	41 (75.9)	
**Comorbid conditions**				
Ischemic cardiomyopathy	87 (43.5)	77 (52.7)	10 (18.5)	< 0.0001
Hypertension	115 (57.5)	87 (59.6)	28 (51.9)	0.4
Atrial fibrillation	52 (26.0)	41 (28.1)	11 (20.4)	0.07
Chronic kidney disease	62 (31.0)	42 (28.8)	20 (37.0)	0.3
Diabetes mellitus	73 (36.5)	55 (37.7)	18 (33.3)	0.7
Prior CABG	35 (17.5)	33 (22.6)	2 (3.7)	0.004
**Medications**				
Beta-blocker	191 (95.5)	140 (95.9)	51 (94.4)	0.9
ACE inhibitor or ARB	175 (87.5)	127 (87.0)	48 (88.9)	0.9
**Loop diuretic dose, mg**				0.9
0	58 (29.0)	42 (28.8)	16 (29.6)	
20–40	90 (45.0)	67 (45.9)	23 (42.6)	
60–80	34 (17.0)	25 (17.1)	9 (16.7)	
> 100	18 (9.0)	12 (8.2)	6 (11.1)	
Digoxin	17 (8.5)	12 (8.2)	5 (9.3)	0.9
Statin	120 (60.0)	95 (65.1)	25 (46.3)	0.02
**Laboratory studies, vital signs and exercise testing**				
Systolic BP, mm Hg	118.0 (104.0–130.0)	120.0 (104.0–130.0)	114.0 (102.8–131.8)	0.7
Sodium, mEq/L	138.0 (137.0 –140.0)	138.0 (137.0–140.0)	138.0 (136.0–140.0)	0.2
Creatinine, mg/dL	1.1 (0.9–1.3)	1.1 (0.91–1.4)	1.0 (0.8–1.2)	0.001
Hemoglobin, g/dL	13.3 (12.3–14.7)	13.9 (12.6–14.9)	12.5 (11.5–13.7)	< 0.0001
GFR, mL/min/1.72 m^2^	67.2 (54.1–84.1)	70.4 (57.7–85.0)	62.5 (52.2–76.0)	0.1
Log(BNP)	2.43 (2.11–28.0)	2.43 (2.19–2.79)	23.1 (1.96–2.83)	0.7
Peak VO_2_, mL/kg/min	14.4 (12.5–15.7)	14.4 (12.8–16.5)	13.9 (11.5–15.0)	0.01
**CMR and echocardiography assessment parameters**				
LVEF, %	24.0 (17.7–30.5)	24.0 (17.6–29.0)	25.1 (18.7–33.1)	0.3
LVEDVI, mL/m^2^	126.3 (102.5–157.0)	128.7 (105.3–162.1)	116.0 (98.4–135.7)	0.04
LVESVI, mL/m^2^	93.7 (73.7–123.6)	95.9 (77.1–127.0)	86.7 (67.2–111.6)	0.06
RVEF, %	37.5 (25.8–45.6)	36.5 (25.7–44.1)	39.3 (25.9–55.1)	0.02
RVEDVI, mL/m^2^	65.8 (52.9–83.1)	66.8 (54.8–83.1)	63.8 (48.0–81.2)	0.1
RVESVI, mL/m^2^	38.8 (29.9–55.5)	39.7 (31.8–56.4)	37.3 (21.2–51.6)	0.05
LGE presence	95 (47.5)	87 (59.6)	8 (14.8)	< 0.0001
CURE-SVD	0.59 (0.45–0.76)	0.61 (0.47–0.77)	0.52 (0.40–0.72)	0.04
**ECG parameters**				
QRS, ms	158 (142–175)	160.0 (140.5–177.5)	155.0 (144.0–164.8)	0.2
QLV, ms	120.0 (87.0–149.3)	110.0 (84.3–145.0)	130.0 (100.0–150.0)	0.3
LBBB	151 (75.5)	103 (70.5)	48 (88.9)	0.01
RBBB	22 (11.0)	21 (14.4)	1 (1.9)	0.02
Paced Rhythm	28 (14.0)	22 (15.1)	6 (11.1)	0.6
**Upgrade or new device**				0.1
*De novo* device	153 (76.5)	107 (73.3)	46 (85.2)	
Upgrade device	47 (23.5)	39 (26.7)	8 (14.8)	
Response measures at 6-months post-CRT				
Fractional change in LVESVI	–0.18 (–0.33 —0.01)	–0.17 (–0.31–0.015)	–0.23 (–0.41—0.07)	0.08
Log(BNP)	2.25 (1.77–2.77)	2.29 (1.80–2.78)	2.14 (1.75–2.58)	0.2
Change in Peak VO_2_, mL/kg/min	0.0 (–1.0–1.2)	–0.025 (–1.4–1.3)	0.11 (–0.54–1.1)	0.6
Survival status at 3 years				0.02
Alive	172 (86.0)	120 (82.2)	52 (96.3)	
Dead	28 (14.0)	26 (17.8)	2 (3.7)	

Values are median (interquartile range) or *n* (%). ACE, angiotensin-converting enzyme; ARB, angiotensin receptor blocker; BMI, body mass index; BNP, B-type natriuretic peptide; BP, blood pressure; CABG, coronary artery bypass graft; CURE-SVD, circumferential uniformity ratio estimate with singular value decomposition; GFR, glomerular filtration rate; LBBB, left bundle branch block; LGE, late gadolinium enhancement; LVEDVI, left ventricular end-diastolic volume index; LVEF, left ventricular ejection fraction; LVESVI, left ventricular end-systolic volume index; NYHA, New York Heart Association; QLV, QRS-LV electrogram time; RBBB, right bundle branch block; RVEDVI, right ventricular end-diastolic volume index; RVEF, right ventricular ejection fraction; RVESVI, right ventricular end-systolic volume index.

### Sex-based differences in clinical parameters, cardiac magnetic resonance findings, and cardiac resynchronization therapy outcomes

**FIGURE 2:**
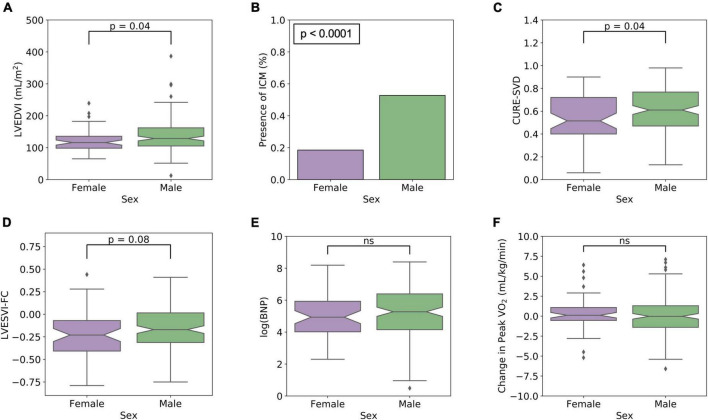
Significant pre-CRT CMR findings and post-CRT response measures stratified only by sex. **(A)** LVEDVI (*p* < 0.04), **(B)** ICM presence (p 0.0001), **(C)** CURE-SVD (*p* = 0.04) are highlighted as these parameters were found to be significantly different between males and females. Compared with males, females were more likely to have smaller LVEDVI, less frequency of ICM, and lower CURE-SVD. In **(D–F)**, the response measures stratified by sex only (as opposed to sex and cardiomyopathy type in Figure 4) are shown to be similar by group.

Significant differences among males (*N* = 146, 73.0%) and females (*N* = 54, 27.0%) were observed for the following baseline clinical parameters and CMR measures: weight (*p* < 0.0001), hemoglobin (*p* < 0.0001), presence of ischemic cardiomyopathy (*p* < 0.0001), presence of LGE (*p* < 0.0001), creatinine (*p* = 0.001), prior CABG (*p* = 0.004), peak VO_2_ (*p* = 0.01), presence of LBBB and RBBB (*p* = 0.01, *p* = 0.02, respectively), RVEF (*p* = 0.02), African-American race (*p* = 0.02), the usage of a statin (*p* = 0.02), LVEDVI (*p* = 0.04), CURE-SVD (*p* = 0.04), and RVESVI (*p* = 0.05). Notched Box and Whisker plots (for LVEDVI and CURE-SVD) are shown in Figures 2A,C and a histogram (for ICM presence) is shown in Figure 2B. Females demonstrated greater RVEF, smaller CMR-based LVEDVI, and more mechanical dyssynchrony based on CURE-SVD; they also had a lower frequency of both LGE and ischemic cardiomyopathy and a higher frequency of LBBB. The three CRT response measures stratified by sex only (as opposed to sex and cardiomyopathy type, as described later) are shown in Figures 2D–F. Median values of the response parameters were more favorable in females (more negative LVESVI-FC, lower post-CRT BNP levels, and higher Δ peak VO_2_), but statistical tests for differences were not apparent without stratification by cardiomyopathy type. A statistically significant difference in time to survival during 3 years of follow-up was present with stratification by sex (*p* = 0.02) as 26 out of the 146 male patients (17.8%) died, but only 2 of the 54 female patients (3.7%) died.

**FIGURE 3:**
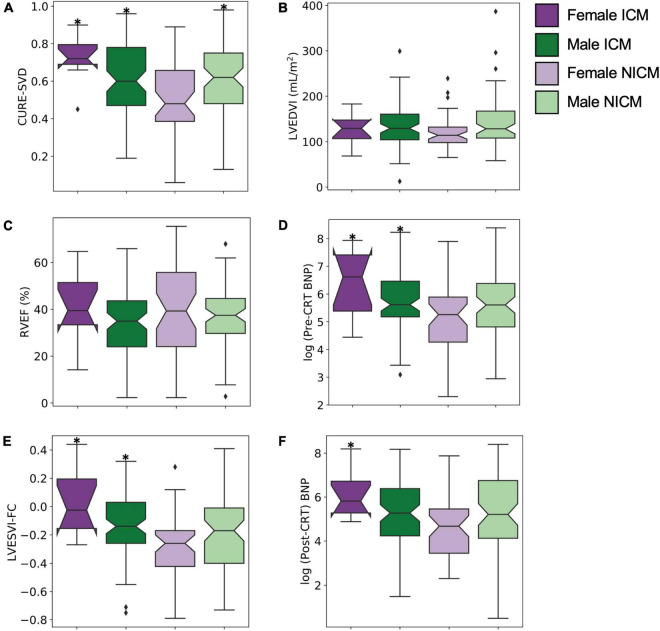
Differences in dyssynchrony, LV size, RV function, hormonal activity, and CRT response measures among cohort stratified by sex and cardiomyopathy type. **(A)** CURE-SVD, **(B)** pre-CRT LVEDVI, **(C)** RVEF, **(D)** log(pre-CRT BNP), **(E)** LVESVI-FC, and **(F)** log(post-CRT BNP) were compared among the four different groups (females with ICM, males with ICM, females with NICM, and males with NICM) using ANOVA. Significant differences between group means were noted for **(A)** CURE-SVD (*p* = 0.003), **(D)** log(pre-CRT BNP) (*p* = 0.01), **(E)** LVESVI-FC (*p* = 0.0006), and **(F)** log(post-CRT BNP) (*p* = 0.05). The Tukey *post hoc* analysis demonstrated differences for pairwise comparisons with *p < 0.05 with the NICM female group as the reference.

**FIGURE 4:**
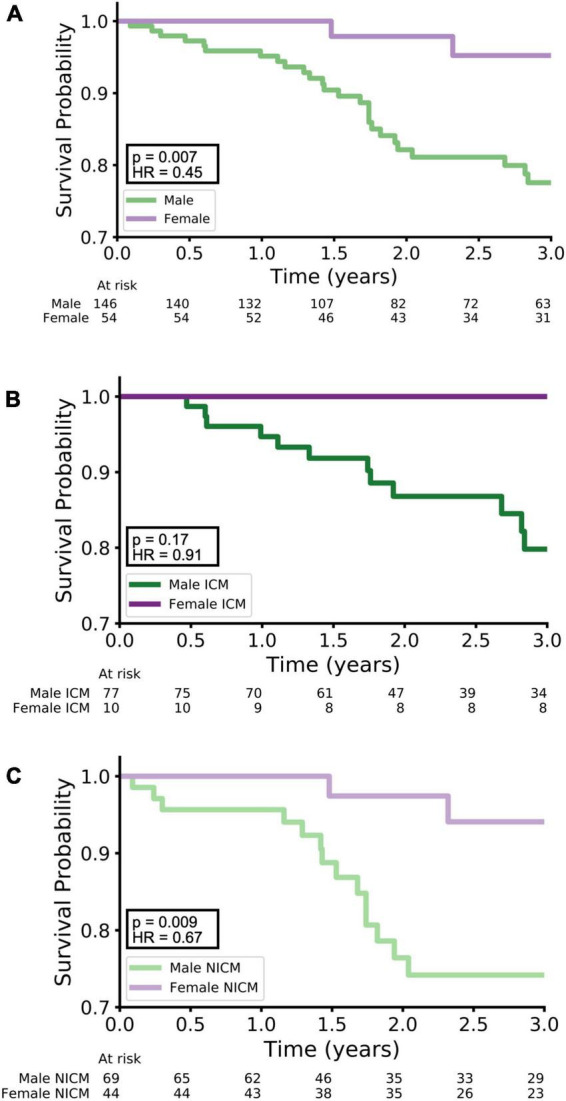
Kaplan–Meier survival curves. Kaplan–Meier curves for survival are shown with stratification by: **(A)** males vs. females, **(B)** males with ICM vs. females with NICM, and **(C)** males vs. NICM versus females with NICM. Females had better survival than males (**A**, *p* = 0.007, HR = 0.45), and females with NICM had better survival than males with NICM (**C**, *p* = 0.009, HR = 0.67). There was no significant difference in survival between females with ICM and males with ICM (**B**, *p* = 0.17, HR = 0.91).

### Linear regression and the interaction of sex and ischemic cardiomyopathy on cardiac resynchronization therapy response measures

The results of the linear regression models are summarized in Table 2. The term representing the interaction of sex and ICM was significant for each of three different models with the following respective dependent variables: CURE-SVD (*p* = 0.004), LVESVI-FC (*p* = 0.008), and log(post-CRT BNP) (*p* = 0.02). The following two observations are highlighted based on these models.

1. In the CURE-SVD model, the effect of being male (sex = 1) decreased CURE-SVD by 0.23 [coefficient of interaction term = -0.23 (CI: –0.39 to –0.076)] when ICM was present, though the ICM coefficient [0.23 (CI: 0.09–0.37)] nearly negated this effect; therefore, for males with ICM (sex = 1, ICM = 1), the only term in the regression model was that of sex [0.11 (CI: 0.03–0.19)] along with the intercept [0.50 (0.44–0.56)]. When ICM was not present in males, a similar relationship was found as the only term remaining in the model was for sex [0.11 (CI: 0.03–0.19)]. Furthermore, the larger effect was observed in females since the female sex (sex = 0) offset both the sex-only term and interaction term, leaving only the ICM term [0.23 (CI: 0.09–0.37)] and intercept. This indicated that females without ICM expressed the lowest CURE-SVD scores.
2. These relationships were similar in the regression models for LVESVI-FC and log(post-CRT BNP). The effect of the female sex drove LVESVI-FC and log(post-CRT BNP) lower, which are both favorable responses, with NICM females having smaller predicted values compared to ICM females; on the other hand, being male resulted in greater predicted LVESVI-FC and log(post-CRT BNP) values regardless of the presence of ICM.

**TABLE 2:** Linear regression models with interaction term.

Model variable	Model coefficient (95% CI)	*P*-value
**(A) CURE-SVD**		
Intercept	0.50 (0.44 to 0.56)	*p* < 0.0001
Sex	0.11 (0.03 to 0.19)	0.006
ICM	0.23 (0.09 to 0.37)	0.001
Sex × ICM	–0.23 (–0.39 to –0.076)	0.004
**(B) LVESVI-FC**		
Intercept	–0.28 (–0.35 to –0.21)	*p* < 0.0001
Sex	0.097 (0.0071 to 0.19)	0.03
ICM	0.30 (0.14 to 0.46)	0.0004
Sex × ICM	–0.25 (–0.43 to –0.067)	0.008
**(C) Log(post-CRT BNP)**		
Intercept	4.7 (4.2 to 5.2)	*p* < 0.0001
Sex	0.60 (–0.017 to 1.2)	0.06
ICM	1.4 (0.33 to 2.6)	0.01
Sex × ICM	–1.5 (–2.7 to –0.25)	0.02
**(D) Δ Peak VO_2_**		
Intercept	0.53 (–0.17 to 1.2)	0.1
Sex	–0.66 (–1.6 to 0.23)	0.1
ICM	–1.4 (–3.0 to 0.21)	0.09
Sex × ICM	1.7 (–0.039 to 3.6)	0.06

### Response measures and survival with stratification by sex and cardiomyopathy type

Figures 3A–F show results for CURE-SVD, pre-CRT LVEDVI, RVEF, log(pre-CRT BNP), LVESVI-FC, and log(post-CRT BNP) with the patients stratified by sex and cardiomyopathy type, effectively dividing the cohort into four groups (ICM males, ICM females, NICM males, and NICM females). Intergroup differences were significant among CURE-SVD (Figure 3A, *p* = 0.003), log-transformed pre-CRT BNP (Figure 3D, *p* = 0.01), LVESVI-FC (Figure 3E, *p* = 0.0006), and log-transformed post-CRT BNP (Figure 3F, *p* = 0.05). The Tukey *post hoc* analysis demonstrated that the CURE-SVD scores of NICM females were lower than those for ICM females along with ICM and NICM males (*p* < 0.05, Figure 3A). The log(pre-CRT BNP) was lower in NICM females compared with that in ICM females and ICM males (*p* < 0.05, Figure 3D). The LVESVI-FC was lower in NICM females compared with that in ICM females and ICM males (*p* < 0.05, Figure 3E). The log(post-CRT BNP) was lower in NICM females compared with that in ICM males (*p* = 0.05, Figure 3F).

Greater QRS duration was associated with improved LVESVI-FC (*p* = 0.008) but not after adjustment for CURE-SVD; QRS duration was not associated with post-CRT BNP levels or change in peak VO_2_ after CRT. Additionally, QRS duration was not significantly different between males and females or among the four phenotypes. RBBB (*p* = 0.03) was associated with suboptimal LVESVI-FC.

The Kaplan–Meier survival curves for three stratifications of the data [(**A**) males vs. females, (**B**) males with ICM vs. females with ICM, and (**C**) males with NICM vs. females with NICM] are shown in Figure 4. Overall, females had better survival than males over 3 years of follow-up with *p* = 0.007 and HR = 0.45 (Figure 4A). While the Cox proportional hazards analysis showed just a borderline improvement in survival for females with ICM relative to males with ICM (*p* = 0.17, HR = 0.91, Figure 4B), females with NICM had a more marked improvement in survival relative to males with NICM (*p* = 0.009, HR = 0.67, Figure 4C).

**FIGURE 5:**
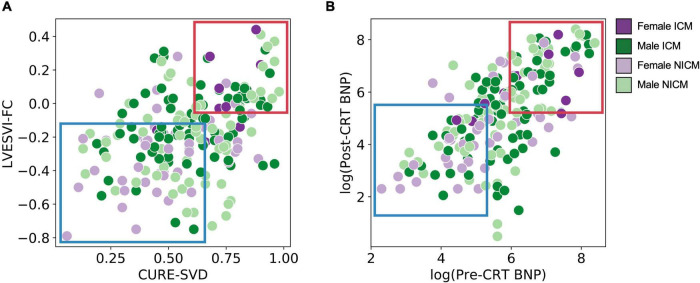
Scatter plots of pre-CRT parameters versus post-CRT response measures for stratified cohort. **(A)** CURE-SVD versus LVESVI-FC for all patients is shown with color and shade based on grouping. Many of the darker points (ICM) appear in the highlighted upper right-hand corner (larger CURE-SVD and positive, unfavorable LVESVI-FC) while many of the lighter points (NICM) appear in the highlighted lower left-hand corner (lower CURE-SVD and negative, favorable LVESVI-FC). Many of the green points (male) appear in the highlighted upper right-hand corner while many of the purple points (female) appear in the highlighted lower left-hand corner. **(B)** log(pre-CRT BNP) versus log(post-CRT BNP) is shown and exhibits similar trends. The plot in panel **(A)** demonstrates clustering of LVESVI-FC and CURE-SVD by sex/cardiomyopathy group, while the plot in panel **(B)** demonstrates differences in BNP by sex/cardiomyopathy group.

Figure 5 presents the possible mechanisms underlying sex-differences in CRT outcomes by illustrating the relationship between the significant pre-CRT parameters and post-CRT response measures. Figure 5A shows a scatterplot of CURE-SVD versus LVESVI-FC, and the points are colored and shaded based on their one of four group assignments. There is a greater proportion of dark purple versus light purple points in the highlighted appear in the highlighted upper right-hand corner (larger CURE-SVD and positive, unfavorable LVESVI-FC), while there is a greater proportion of light purple versus dark purple points in the highlighted lower left-hand corner (lower CURE-SVD and negative, favorable LVESVI-FC). This indicates that ICM played a role in LVESVI-FC. Furthermore, many of the green points appear in the highlighted upper right-hand corner while many of the purple points appear in the highlighted lower left-hand corner; this demonstrates the role of sex in LVESVI-FC and suggests that the mechanism for more favorable responses in females is their smaller CURE-SVD scores. The scatterplot of log-transformed pre-CRT BNP levels versus log-transformed post-CRT BNP levels shown in Figure 5B exhibited similar trends. This graph suggested that sex plays a role in post-CRT BNP levels and that the mechanism for more favorable responses in females is their smaller pre-CRT BNP levels.

## Discussion

### Summary of differences in outcomes after cardiac resynchronization therapy by sex

The analysis based on an observational study with CMR provides novel insights into possible mechanisms previously unexplored with respect to CRT outcomes in males versus females.

1. Females had smaller CMR-based left ventricular end-diastolic volume indices and more dyssynchrony based on the CURE-SVD determined with DENSE.
2. Females were less likely to have ischemic cardiomyopathy and had less LGE compared with males.
3. Females had more favorable right ventricular ejection fractions and were more likely to have LBBB.
4. Females had greater improvements in LV function after CRT.
5. Females had a better 3-year survival probability.

### Cardiac magnetic resonance-derived CURE-SVD and B-type natriuretic peptide explain sex-differences in cardiac resynchronization therapy outcomes

The CURE-SVD measures the extent of simultaneous contraction (negative circumferential strain) and stretch (positive circumferential strain) in LV segments using CMR (23). It ranges between 0 and 1, and values closer to 0 indicate greater dyssynchrony. In our present study, females generally had lower CURE-SVD (and thus more dyssynchrony) than males while the female NICM group had the lowest CURE-SVD among all the groups. Our group has previously demonstrated the effectiveness of CURE-SVD in predicting LV functional improvement (LVESVI-FC) in CRT within prior cohorts and has shown that a lower CURE-SVD correlates with a more favorable LVESVI-FC (23–27); however, CURE-SVD’s association with female sex is a new finding. This association provides a possible mechanistic insight as to why females are more likely to have more beneficial outcomes after CRT – they have greater degrees of dyssynchrony and consequently experience more favorable changes in LV functional improvement from ventricular resynchronization. Additionally, in the NICM female group, an even more favorable LVESVI-FC was observed due to both a lower CURE-SVD and the absence of scar from prior myocardial infarction. Our group has previously shown that ICM decreases the success of CRT as non-conductive scar from prior myocardial infarction hinders travel of the paced electrical impulses. Therefore, because females are more likely to have a lower CURE-SVD and less likely to have ICM, they respond better to CRT.

Another interesting finding concerned neurohormonal activity measured as the logarithm of serum BNP levels before CRT implantation. In our current study, we report that the female NICM group had lower levels of pre-CRT BNP compared with the male ICM and female ICM groups. This is rather striking as other studies in non-CRT cohorts have shown that BNP levels are lower in males than in females and increase with age (28, 29). Incidentally, age was not different between the male and female groups within our cohort. Furthermore, obesity, renal disease and kidney dysfunction are known to influence BNP levels (30, 31), yet these parameters were not significant among sex within our cohort. We along with others have shown that lower levels of BNP are correlated with better CRT response (19, 27), and since in our current cohort, and as the NICM females in our cohort had the lowest BNP levels, this measure may mechanistically explain the better outcomes of NICM females. Still, our findings of lower BNP levels in females with no difference in age or kidney function coupled with favorable outcomes warrant further investigation.

### Cardiac magnetic resonance phenotypes for female non-ischemic and ischemic cardiomyopathies

These findings lead to interesting observations regarding the underlying mechanisms of the observed sex-based differences in CMR findings and CRT response measures in this study. In particular, we have shown that a particular phenotype of female non-ischemic cardiomyopathy was characterized by a more favorable CURE-SVD (more dyssynchrony) at baseline and lower baseline pre-CRT BNP levels. We also showed that this phenotype exhibited more favorable responses to CRT with a more favorable LVESVI-FC and lower log(post-CRT BNP). Taken together, these findings suggest that sex-related differences in CMR-derived CURE-SVD and BNP serum levels define distinct female ischemic and non-ischemic cardiomyopathy phenotypes and may explain differences in survival and outcome, although these findings must be interpreted in the context of the observational study design, which establishes associations rather than causal relationships. Furthermore, the CURE-SVD parameter has proven to be a robust tool in predicting CRT response and may be worth considering during patient selection for the therapy, as we have shown that it reliably predicts response, survival, and arrhythmia risk in patients undergoing CRT implants, even after adjustment for clinical risk models (23–25). A primer for DENSE and CURE-SVD for cardiologists is also available as Supplementary material in a recent publication (24).

### Importance of sex in cardiac resynchronization therapy guidelines and future clinical trials

The results of this study highlight the importance of considering the impact of a patient’s sex and cardiomyopathy etiology when evaluating the patient for CRT. After adjustment by cardiomyopathy etiology, male patients had less favorable outcomes and lower, which may be an important consideration in the timely allocation of evidence-based medical therapies in patients with heart failure undergoing CRT. Our study also underscores the need for the inclusion of more females in CRT research studies and clinical trials; females evidently differ in their response to CRT, and more information may be helpful in further specifying CRT treatment based on sex in the context of cardiomyopathy etiology.

### Limitations

Our study cohort included 54 females out of a total of 200 patients (27.0%), and only 10 of those females had ICM. A larger number of females may strengthen the connections that we observed between sex, CMR finding, and CRT outcomes. We also acknowledge that medical therapy for heart failure continues to evolve (i.e., more patients are being prescribed sodium-glucose cotransporter-2 inhibitors and angiotensin-neprilysin inhibitors). Longitudinal studies of outcomes for devices in heart failure with long-term follow-up may lag behind adoption of novel medical therapies, and sex-based differences in outcomes may change as medical therapies change. For example, angiotensin-neprilysin inhibitors have been shown to increase BNP expression (32). We acknowledge the potential for bias in any cohort study. In this study, we wanted to minimize that chance that analysis of response measures would be influenced by a knowledge of patient outcomes. We did this by ensuring that response measures were determined independently without knowledge of patient outcomes. Finally, our cohort underwent traditional CRT implants with defibrillators, and we did not consider patients with conduction system pacing; however, an evaluation of sex-related differences in parameters of interest in a cohort of patients with conduction system pacing is planned in the future.

## Data availability statement

The datasets presented in this article are not readily available because use of the dataset is restricted to users at the author’s institution. Requests to access the datasets should be directed to KB, bilchick@virginia.edu.

## Ethics statement

The studies involving human participants were reviewed and approved by the University of Virginia Institutional Review Board for Human Subjects Research. The patients/participants provided their written informed consent to participate in this study.

## Author contributions

DB and KB contributed to the study conception, data analysis, and writing of the manuscript. ST, MA, PO, and XG contributed to the data analysis and a critical review of the manuscript. RM, AD, OM, JM, and PM contributed to the study conception and enrollment of patients. SM, MS, CK, FE, and JH contributed to the critical review of the manuscript. PO and FE contributed to the study conception. All authors contributed to the article and approved the submitted version.
