# Supplementary material for: Cardiac magnetic resonance defines mechanisms of sex-based differences in outcomes following cardiac resynchronization therapy
Source: Front Cardiovasc Med. 2022 Sep 15;9:1007806. doi: 10.3389/fcvm.2022.1007806 (PMC9521735; doi:10.3389/fcvm.2022.1007806)
Supplement: Supplementary file 1 [file Table_1.DOCX]

**Supplementary Material: Statistical Methods**

**The Expectation-Maximization (E-M) Algorithm**

We considered CRT response as a composite of three metrics: change in peak VO_2_, the LVESVI-FC, and log-transformed BNP. In cases of composite outcomes, maximum likelihood estimation (MLE) works well in imputing missing values. The expectation-maximization (EM) algorithm for matrix completion uses a robust maximum likelihood estimator. We used this algorithm to impute the missing response measures as it iteratively optimizes the covariance response matrix and mean vector until it converges, which gives a maximum likelihood estimate for the missing values. Briefly, the missing post-CRT peak VO_2_ values were imputed while also taking into consideration the non-missing LVESVI-FC and BNP response values.

In order to prove the robustness of the imputation algorithm, we constructed two linear regression models (with the same independent parameters shown previously to be significant with respect to peak VO_2_) for the Δ Peak VO_2_ response measure: (1) one using only patients with measured VO_2_ before and after CRT (n=153; 76.5% of cohort) and (2) one using the complete, imputed data set. The model coefficients for the two models were very close (within 10%), and the p-values were also very similar. A table of these findings is shown below. Certainly, the additional patients in the full cohort (model 2) would be expected to account for small differences in the model coefficients. Overall, this confirms that our VO_2_ findings are reliable with the method of imputation.

| **Multivariable Linear Regression Model for Δ Peak VO_2_:** | | | | |
| --- | --- | --- | --- | --- |
| **Complete Case Analysis (153/200) vs Original Analysis (Imputed Data, 200/200)** | | | | |
| **Model Variable** | **Standardized Coefficient (Complete Case Analysis)** | **Standardized Coefficient (Original Analysis)** | **p Value  (Complete Case Analysis)** | **p Value  (Original Analysis)** |
| Intercept | 0 | 0 | 0.01 | 0.03 |
| Peak VO_2_ at Baseline | -0.337 | -0.287 | <0.0001 | <0.0001 |
| GFR | 0.3 | 0.254 | 0.0001 | 0.0002 |
| SBP | -0.163 | -0.146 | 0.03 | 0.03 |
